# Sustained effects of auricular point acupressure on chemotherapy-induced neuropathy: a randomized controlled trial follow-up study

**DOI:** 10.1007/s11764-026-02104-3

**Published:** 2026-09-01

**Authors:** Nada Lukkahatai, Yannuo Jiang, Jingyu Zhang, Jennifer Kawi, Hulin Wu, Hongyu Wang, Claudia M. Campbell, Johannes Thrul, Salahadin Abdi, Eileen D. Hacker, Constance M. Johnson, Thomas J. Smith

**Affiliations:** 1 Johns Hopkins University School of Nursing, 525 North Wolfe Street, Baltimore, MD 21205, USA; 2 Sidney Kimmel Comprehensive Cancer Center at Johns Hopkins, Baltimore, MD, USA; 3 School of Public Health, The University of Texas Health Science Center at Houston, Houston, TX, USA; 4 Cizik School of Nursing, The University of Texas Health Science Center at Houston, Houston, TX, USA; 5 McGovern Medical School, The University of Texas Health Science Center at Houston, Houston, TX, USA; 6 Department of Psychiatry and Behavioral Medicine, Johns Hopkins University School of Medicine, Baltimore, MD, USA; 7 Johns Hopkins Bloomberg School of Public Health, Baltimore, MD, USA; 8 Department of Pain Medicine, Division of Anesthesiology, Critical Care & Pain Medicine, The University of Texas MD Anderson Cancer Center, Houston, TX, USA; 9 Department of Nursing, The University of Texas MD Anderson Cancer Center, Houston, TX, USA; 10 Johns Hopkins University School of Medicine, Baltimore, MD, USA

**Keywords:** Chemotherapy-induced neuropathy, Auricular point acupressure, Acupressure, Auriculotherapy, Randomized controlled trial

## Abstract

**Purpose:**

Chemotherapy-induced neuropathy (CIN) is a persistent condition that impairs function and quality of life. Auricular point acupressure (APA) has shown short-term benefit for CIN, but the durability of these effects after treatment completion is unknown. This study evaluated the sustainability of symptom improvements for 3 months following a 4-week APA intervention.

**Methods:**

This prespecified secondary analysis of a randomized wait-list controlled trial compared mobile-supported APA (mAPA) and virtual APA (vAPA) in adults with moderate or greater CIN who received APA during the initial treatment phase (mAPA, *n* = 80; vAPA, *n* = 75). Outcomes at 1, 2, and 3 months post-intervention were analyzed using generalized estimating equations with multiple imputation. The primary outcome was CIN severity, measured with an individualized composite outcome (ICO); the secondary outcome was CIN interference.

**Results:**

Reductions in CIN severity and interference were maintained throughout follow-up in both groups (all *p* < .001). ICO scores decreased by 3.10, 3.36, and 3.61 points in mAPA and by 2.88, 2.90, and 3.21 points in vAPA at 1, 2, and 3 months, respectively. Benefits were maintained post-treatment, with higher retention in the mAPA group.

**Conclusions:**

Improvements in CIN severity and interference after APA were sustained for up to 3 months post-treatment, suggesting a durable benefit as a self-management strategy. Larger studies with longer follow-up and untreated comparison groups are needed.

**Implications for cancer survivors:**

APA may offer survivors a durable, self-administered, nonpharmacologic option for managing CIN well beyond active treatment, without requiring ongoing clinical visits.

**Trial registration:**

ClinicalTrials.gov, ID NCT04920097 registered on 3 June 2021.

## Introduction

Chemotherapy-induced neuropathy (CIN) remains a persistent and clinically significant challenge among cancer survivors, often extending well beyond the completion of treatment. Symptoms typically emerge during chemotherapy and may peak within several months after treatment completion, followed by gradual improvement in some individuals. However, recovery is often incomplete, and a substantial proportion of patients continue to experience chronic neuropathic symptoms, including pain, numbness, and tingling, that interfere with daily functioning and quality of life [1, 2]. Long-term evidence further highlights the durability of this burden. In a multicenter study of colorectal cancer survivors treated with oxaliplatin, 31% of patients continued to experience chemotherapy-induced peripheral neuropathy up to five years after treatment, with minimal improvement over time and a substantial proportion reporting persistent neuropathic pain and reduced quality of life [3]. These findings suggest that CIN is not merely a transient treatment-related toxicity but, for many patients, a long-lasting condition with significant physical and psychological consequences. Despite clinical guidelines, effective long-term management remains limited, further emphasizing the need for sustainable, patient-centered interventions that extend beyond short-term symptom relief [4, 5].

Auricular point acupressure (APA), a noninvasive modality derived from auricular therapy, has been explored as a nonpharmacologic approach for symptom management in both chronic pain and cancer populations. APA involves stimulation of specific ear points associated with neurological and somatic pathways that modulate pain and regulate symptoms, allowing patients to self-administer treatment outside of clinical settings. Prior studies suggest that APA is feasible, safe, and may be associated with improvements in symptoms such as pain, fatigue, and sleep disturbance [6–10]. Pilot studies in patients with CIN also demonstrated improvements in pain, numbness, tingling, sensory thresholds, inflammatory biomarkers, and brain functional connectivity following APA interventions [10, 11]. Building on this body of work, we conducted a randomized controlled trial to evaluate the short-term effects of APA for CIN. Participants were randomized to either an in-person APA intervention with mobile support (mAPA), a virtual self-guided APA intervention delivered through a smartphone application (vAPA), or usual care as control. The mAPA intervention included in-person seed placement and training, smartphone instructional support, and Zoom-based coaching, whereas the vAPA intervention consisted of self-guided instructional videos and remote coaching delivered through the smartphone application. The intervention was generally well tolerated, with high adherence and minimal adverse events. The full primary outcome findings from the parent randomized controlled trial evaluating the immediate effects of the 4-week APA intervention on CIN symptoms and physical function will be reported separately [12].

An important remaining question is whether these effects are sustained over time. Outcomes assessed immediately after treatment completion may, in part, reflect nonspecific influences such as treatment expectation, attention, or engagement with the intervention, particularly in nonpharmacologic and behavioral interventions where expectancy-related effects can contribute to perceived symptom improvement [13]. As a result, improvements observed at the end of the intervention may not fully represent the stability or durability of treatment effects. Examining outcomes beyond the active intervention period is therefore important to determine whether these improvements are maintained, continue to evolve, or diminish over time.

To address this gap, this secondary analysis examined the sustainability of the effects of mAPA and vAPA following intervention completion. Specifically, we evaluated whether improvements in CIN symptom severity and physical function were maintained during the three-month follow-up period and whether additional changes occurred after treatment ended. This follow-up analysis was prepared as a separate report because it addresses a distinct durability question that was not the focus of the parent trial’s immediate post-intervention efficacy analysis.

## Methods

### Study design

This study is a prespecified secondary analysis of a randomized controlled design evaluating APA for CIN. The parent trial employed a three-arm randomized wait-list controlled design comparing mAPA, vAPA, and usual care in oncology outpatient settings in Baltimore, Maryland, and Houston, Texas between July 2021 and October 2024. Detailed descriptions of the study design and intervention procedures have been reported elsewhere [14].

The primary endpoint analysis, which focused on the immediate efficacy of APA during the 4-week randomized treatment phase, is reported in a separate manuscript [12] because it addresses the parent trial’s main comparative efficacy question among mAPA, vAPA, and usual care. The present manuscript addresses a distinct secondary question: whether improvements observed after APA were sustained after the active intervention ended. Because participants assigned to usual care were offered APA after completion of the 4-week control period for ethical and pragmatic reasons, the follow-up phase did not include an untreated concurrent control group. Therefore, the present analysis was restricted to participants who received mAPA or vAPA during the initial randomized treatment phase and focuses on post-treatment symptom trajectories, durability of response, retention, and clinical relevance. This separate reporting allows the sustainability findings to be presented with appropriate methodological detail and interpretive caution.

### Study participants

Adults (≥ 18 years) with CIN were recruited from oncology outpatient clinics. Eligible participants had received neurotoxic chemotherapy (e.g., platinum-based agents, taxanes, vinca alkaloids, bortezomib, or eribulin) and reported moderate or greater CIN-related symptoms, defined as a score ≥ 4 for pain, numbness, or tingling on an 11-point numerical rating scale. Participants with pre-existing peripheral neuropathy that worsened after chemotherapy were also eligible. Key exclusion criteria included concurrent use of investigational pain therapies, prior neurolytic procedures for pain control, and known allergies to adhesive materials. Full eligibility criteria have been described previously [14]. Participants with pre-existing peripheral neuropathy were eligible only if their neuropathy symptoms worsened after neurotoxic chemotherapy, consistent with the study’s focus on clinically meaningful CIN-related symptom burden.

### Randomization and allocation concealment

Participants were randomized in a 1:1:1 ratio to mAPA, vAPA, or usual care using a computer-generated schedule with allocation concealment. Additional details regarding randomization procedures are reported elsewhere [14]. The final group sizes were not identical because of the completed randomization sequence and participant flow at study closure; the planned allocation ratio remained 1:1:1.

### Blinding

Due to the nature of the intervention, participants could not be blinded to the assignment to the APA or control condition, nor the intervention delivery modality (mAPA vs. vAPA). However, participants were blinded to the main study hypothesis regarding the expected effectiveness of intervention. Study personnel involved in data collection and analysis as well as multiple principal investigators (MPIs) were blinded to group assignments. Additional details on blinding procedures have been reported previously [14].

### Intervention

Participants were randomized to in-person with mAPA, vAPA, or usual care. Both APA interventions involved stimulation of predefined auricular points using ear seeds applied for 4 weeks, with participants instructed to perform daily self-stimulation. The mAPA group received initial in-person training followed by virtual reinforcement, whereas the vAPA group used standardized instructional videos and remote guidance through a mobile application. Participants in the usual care group completed symptom monitoring and were offered APA after the control phase. Additional details regarding intervention procedures, training, and fidelity monitoring have been reported previously [14]. In brief, ear seeds were applied to predefined auricular points, participants were taught to press the seeds daily, and intervention support focused on correct stimulation, symptom monitoring, troubleshooting, and reinforcement of self-management.

### Measures

Baseline demographic and clinical characteristics included age, sex, race, ethnicity, education, employment status, cancer diagnosis, years since diagnosis, medication and supplement use, comorbidity burden, and functional status. Functional status was assessed using the Eastern Cooperative Oncology Group (ECOG) Performance Status scale, a clinician-rated 6-point ordinal measure ranging from 0 (fully active) to 5 (dead), with higher scores indicating worse functional performance. ECOG scores were categorized as 0–1 (preserved functional status) and ≥ 2 (reduced functional status) [15]. Neurotoxicity-related physical and functional well-being was assessed using the Functional Assessment of Cancer Therapy/Gynecologic Oncology Group–Neurotoxicity (FACT/GOG-Ntx) subscale, an 11-item measure scored from 0 to 44, with higher scores indicating better function and less neurotoxicity-related impairment [16].

The primary outcome for this secondary analysis was CIN symptom severity, operationalized using an individualized composite outcome (ICO). The ICO was derived from the Modified Brief Pain Inventory–Chemotherapy-Induced Neuropathy (BPI-CIN) [10], which assesses pain, numbness, tingling, and stiffness across multiple body regions using 0–10 numerical rating scales. For each participant, the ICO was defined as the highest baseline score across all symptom-location combinations to capture the most severe CIN manifestation [17, 18]. Secondary outcomes included pain interference (BPI-I) and selected measures of physical function and quality of life. Detailed descriptions of all outcome measures collected in the parent trial have been reported previously [12, 14]. These secondary outcomes were included to help determine whether sustained symptom reduction was accompanied by improvements in interference, function, and patient well-being.

For the present analysis, outcomes assessed at baseline, 4 weeks (completion) 1, 2, and 3 months following completion of the intervention were used to evaluate maintenance of post-treatment changes over time.

### Data collection

Data were collected and managed using a single Research Electronic Data Capture (REDCap) system hosted at Johns Hopkins University [19, 20]. Assessments were conducted at baseline, weekly during the 4-week intervention period, immediately post 4-weekintervention, and at 1, 2, and 3 months following completion of the intervention. Detailed data collection procedures, quality control processes, and data management protocols have been reported previously [14].

### Statistical analysis

Analyses included all participants who received mAPA or vAPA during the initial randomized treatment phase, with a significant threshold of α = 0.05. Because participants in the usual care group were re-randomized to receive APA after the initial 4-week period, no untreated control group was available during follow-up; therefore, analyses were restricted to participants who received APA during the initial treatment phase and focused on within-participant changes over time. Post-treatment outcomes at 1, 2, and 3 months (M1, M2, and M3) were analyzed using generalized estimating equations (GEE) to account for correlations among repeated measurements within individuals. Two reference time points were used to characterize post-treatment trajectories: (1) baseline (Week 1) to evaluate continued improvement over time and (2) end of treatment (Week 4) to evaluate maintenance of post-treatment effects.

Outcomes demonstrating improvement at Week 4 were evaluated for maintenance during follow-up, whereas outcomes without apparent post-treatment improvement were explored descriptively for potential delayed changes over time. Both continuous outcomes (e.g., ICO and BPI scores) were analyzed. Missing data were addressed using multilevel multiple imputation methods incorporating baseline characteristics and outcome variables. Imputed datasets were analyzed using GEE, and parameter estimates were combined using standard pooling procedures. Given the absence of an untreated control group during follow-up, findings should be interpreted as descriptive of post-treatment trajectories rather than causal estimates of sustained APA effects.

## Results

### Participant flow

The detailed participant flow and enrollment procedures for the parent randomized controlled trial are described elsewhere [12]. Briefly, 227 participants were randomized to mAPA (*n* = 80), vAPA (*n* = 75), or usual care (*n* = 72). For the present secondary analysis, only participants who received APA during the initial randomized treatment phase were included because participants in the usual care group were offered APA after the 4-week control period as part of the wait-list design. The analytic sample therefore included 155 participants (mAPA, *n* = 80; vAPA, *n* = 75). Follow-up completion rates at 1, 2, and 3 months post-intervention were 62.5%, 51.3%, and 48.8% for mAPA and 34.7%, 30.7%, and 25.3% for vAPA, respectively (Fig. 1). Retention was consistently higher in the mAPA group than in the vAPA group across all follow-up assessments.

The most common reasons for missing follow-up data included loss to follow-up, lack of time or commitment, unmet expectations regarding intervention efficacy, and medical-related issues, with higher attrition observed in the vAPA group. Participants with missing follow-up data generally demonstrated similar baseline CIN severity and pain interference scores compared with retained participants. Missingness was more common in the vAPA group than in the mAPA group and was addressed using multiple imputation and sensitivity analyses.

### Baseline characteristics

Baseline demographic and clinical characteristics were generally comparable between the mAPA and vAPA groups, with no statistically significant between-group differences (Table 1). Mean age was 65.10 years (SD = 9.89) in the mAPA group and 62.47 years (SD = 12.72) in the vAPA group. Participants were predominantly female and White and gastrointestinal and breast cancers were the most commonly reported cancer type. Participants in both groups reported moderate-to-severe baseline CIN symptoms, with similar individualized composite outcome (ICO) scores (mAPA: mean = 8.01, SD = 1.68; vAPA: mean = 8.00, SD = 1.81) and pain interference scores at study entry. Baseline functional status and comorbidity burden were also comparable between groups. Most participants demonstrated good functional performance based on ECOG scores, although a subset reported impaired or frail functional status. Use of prescription medications, over-the-counter medications, and supplements for pain and sleep management was similar across intervention groups.

### Primary outcome: CIN severity (ICO)

Changes in CIN severity during the post-intervention follow-up period are presented in Table 2 and Fig. 2. Compared with baseline, significant reductions in ICO scores were sustained throughout the 3-month follow-up period in both intervention groups. In the mAPA group, mean ICO scores decreased by 3.10 points at 1 month (Estimate = − 3.10, 95% CI −3.78 to −2.41, *p* < 0.001), 3.36 points at 2 months (Estimate = − 3.36, 95% CI −4.15 to −2.57, *p* < 0.001), and 3.61 points at 3 months (Estimate = − 3.61, 95% CI −4.42 to −2.79, *p* < 0.001) relative to baseline. Similarly, in the vAPA group, mean ICO scores decreased by 2.88 points at 1 month (Estimate = − 2.88, 95% CI −3.67 to −2.08, *p* < 0.001), 2.90 points at 2 months (Estimate = − 2.90, 95% CI −3.63 to −2.17, *p* < 0.001), and 3.21 points at 3 months (Estimate = − 3.21, 95% CI −4.04 to −2.38, *p* < 0.001) relative to baseline. Significant reductions in CIN interference scores were also sustained throughout the follow-up period in both groups (all *p* < 0.001). Individual upper- and lower-extremity symptom severity scores (numbness, pain, stiffness, and tingling) followed a broadly similar trajectory, with the largest reductions from baseline occurring by the end of the active intervention period and improvements generally persisting, with some fluctuation in upper-extremity symptoms, through the 3-month follow-up (Fig. 2, panels C–D). Overall, reductions in CIN severity observed following the intervention were sustained throughout the follow-up period, with no evidence of symptom worsening after treatment completion. Numerical improvements continued over time in both groups, particularly among participants receiving mAPA. Patterns of findings were generally consistent in sensitivity analyses using complete-case data without multiple imputations.

### Secondary outcome: CIN-interference

Changes in CIN interference during the post-intervention follow-up period are presented in Table 2 and Fig. 2, panel B. Compared with baseline, significant reductions in CIN interference were maintained throughout the 3-month follow-up period in both intervention groups. In the mAPA group, mean CIN interference scores decreased by 1.30 points at 1 month (Estimate = − 1.297, SE = 0.255, *p* < 0.001), 1.36 points at 2 months (Estimate = − 1.358, SE = 0.252, *p* < 0.001), and 1.60 points at 3 months (Estimate = − 1.600, SE = 0.256, *p* < 0.001) relative to baseline. Similarly, in the vAPA group, mean CIN interference scores decreased by 1.18 points at 1 month (Estimate = − 1.182, SE = 0.254, *p* < 0.001), 1.22 points at 2 months (Estimate = − 1.217, SE = 0.278, *p* < 0.001), and 1.28 points at 3 months (Estimate = − 1.281, SE = 0.283, *p* < 0.001) relative to baseline. Overall, improvements in CIN interference observed following the intervention were sustained throughout the follow-up period, with no evidence of worsening symptoms after treatment completion. Although the present follow-up analysis did not emphasize broader quality-of-life outcomes, sustained improvements in CIN interference provide patient-centered evidence that symptom reductions may have affected daily well-being. CIN interference captures the impact of symptoms on daily activity, mood, walking ability, work, relationships, sleep, and enjoyment of life, and therefore helps contextualize the clinical relevance of the observed improvements.

### Sensitivity analysis

Sensitivity analyses using complete-case data without multiple imputation demonstrated patterns generally consistent with the primary analyses. Significant reductions in CIN severity and CIN interference observed following the intervention were maintained throughout the follow-up period in both intervention groups (Supplementary Table S1). The magnitude and direction of changes were similar to those observed in the imputed analyses, with no evidence of symptom worsening during follow-up. Outcomes that did not demonstrate significant effects immediately following the intervention similarly showed no consistent evidence of delayed-onset improvement during the follow-up period. Patterns of findings were generally consistent between combined and treatment-specific analyses.

## Discussion

This secondary analysis examined the sustainability of APA following completion of a 4-week intervention for CIN. The findings demonstrated that improvements in both CIN severity and CIN interference were maintained for up to three months after intervention completion in participants receiving either mobile-supported APA (mAPA) or virtual APA (vAPA). Importantly, symptom improvements persisted after the active 4-week intervention ended, with no evidence of symptom worsening during follow-up. These findings suggest that APA may provide durable benefits for individuals experiencing chronic CIN and support its potential role as a sustainable self-management strategy for cancer survivors. Given the chronic and often refractory nature of CIN, sustained symptom relief following a brief, self-administered intervention is clinically meaningful and aligns with growing evidence supporting APA as a nonpharmacologic symptom management strategy across chronic conditions [9, 21, 22].

The sustained reduction in CIN severity is particularly noteworthy given the persistent nature of chemotherapy-induced neuropathy. Although some recovery may occur after completion of neurotoxic chemotherapy, symptoms frequently persist for months or years, and many cancer survivors continue to experience chronic neuropathic symptoms that interfere with daily functioning and quality of life [3–5, 23]. Longitudinal studies have reported that a substantial proportion of patients continue to experience neuropathy years after treatment completion, highlighting the limited spontaneous recovery observed in many survivors [3]. In the present study, participants in both intervention groups maintained reductions of approximately three points in ICO scores relative to baseline throughout the follow-up period. These findings suggest that the symptom improvements observed immediately following APA treatment were not transient and may extend beyond the period of active intervention.

The sustained improvements observed in this study are consistent with previous APA research in chronic symptom populations. In a randomized controlled trial of older adults with chronic low back pain, Kawi et al. (2025) reported that improvements in pain and physical function following a four-week APA intervention were maintained for up to six months after treatment completion. Similar to the present study, participants did not receive additional APA treatments during follow-up, yet treatment benefits persisted well beyond the intervention period. Earlier APA studies have also demonstrated improvements in chronic pain, fatigue, sleep disturbance, and symptom burden among diverse populations, supporting the potential for lasting benefits beyond active treatment [21, 24, 25]. Although the populations and outcomes differed, these studies collectively suggest that APA may produce benefits that remain evident after active treatment has ended. Notably, the trajectory of improvement differed somewhat between studies. Whereas improvements in chronic low back pain were primarily maintained over time, participants in the present study demonstrated continued numerical improvement in CIN severity and interference during follow-up. This pattern may reflect the gradual recovery process often observed in neuropathic conditions and warrants further investigation.

The present findings are also consistent with evidence from auricular acupuncture studies conducted in patients with diabetic peripheral neuropathy (DPN), another chronic neuropathic condition characterized by sensory symptoms, pain, and functional impairment. Several studies have reported that auricular acupuncture can significantly reduce neuropathic pain intensity, improve sensory symptoms, and enhance quality of life among individuals with DPN [26, 27]. In a randomized controlled trial, Mohammadalinia et al. (2025) found that participants receiving auricular acupressure experienced clinically meaningful reductions in neuropathic symptoms that persisted beyond the treatment period [26]. Although DPN and CIN differ in etiology, both involve peripheral nerve damage and share common symptoms, including numbness, tingling, burning sensations, and pain. The sustained symptom improvements observed in the current APA study parallel findings from auricular acupressure studies in DPN, suggesting that stimulation of auricular points may provide durable symptom relief across different neuropathic conditions.

Improvements in CIN interference were also maintained throughout the follow-up period. Participants in both intervention groups reported significantly lower interference scores at all follow-up assessments compared with baseline, suggesting that symptom improvements may be translated into sustained benefits in daily functioning. Because CIN often affects mobility, balance, fine motor function, and participation in everyday activities, reducing symptom interference may be particularly meaningful from a patient-centered perspective [4, 5]. Similar functional improvements have been reported in studies of auricular acupressure for diabetic neuropathy, where improvements in physical functioning and quality of life accompanied reductions in neuropathic symptoms [26]. The magnitude of improvement is clinically interpretable because both ICO and BPI-CIN interference use 0–10 scoring; reductions of approximately 1 to 3 points may represent meaningful changes in symptoms and daily activity interference for patients. However, because this follow-up analysis did not include a concurrent untreated control group and relied primarily on patient-reported outcomes, these findings should be interpreted as preliminary evidence of patient-centered benefit rather than definitive evidence of functional recovery.

An important observation was that both mAPA and vAPA demonstrated sustained improvements throughout the follow-up period. This finding suggests that APA may remain beneficial when delivered through either an in-person supported model or a predominantly self-guided virtual format. Such flexibility has important implications for accessibility and scalability, particularly for cancer survivors who may have limited access to specialty supportive pain care services. The increasing emphasis on remote symptom management and digital health interventions in cancer survivorship further underscores the relevance of these findings [28, 29]. However, retention rates during follow-up were substantially higher in the mAPA group than in the vAPA group. Although symptom trajectories appeared generally similar between groups, the higher attrition observed in the virtual intervention highlights the challenges of maintaining engagement in fully remote symptom management programs. The higher retention in the mAPA group may reflect the added structure and interpersonal support provided by the initial in-person session, including direct ear seed placement, hands-on training, and early relationship-building with study staff. In contrast, the vAPA group relied more heavily on self-guided use of the mobile application and remote support, which may have required greater participant motivation, technology comfort, and self-management capacity. This pattern is consistent with prior digital health literature indicating that remotely delivered interventions often experience substantial attrition and that engagement improves when interventions include human support, reminders, or other features that sustain participant involvement [30–32]. Hybrid models that combine remote delivery with structured check-ins or brief interpersonal support may help preserve accessibility while improving retention.

The findings also have important clinical implications. Management options for established CIN remain limited, and current clinical guidelines identify duloxetine as the only pharmacologic therapy with sufficient evidence to support its use for painful CIN [4, 5]. Even then, treatment benefits are generally modest, and many patients continue to experience residual symptoms. Recent systematic reviews have concluded that evidence supporting most pharmacologic and nonpharmacologic interventions for CIN remains limited or inconsistent [5, 33]. APA represents a low-risk, noninvasive, and self-administered intervention that can be incorporated into survivorship care with minimal resource requirements. Compared with other nonpharmacological intervention programs, APA may offer additional advantages in terms of accessibility, cost, and patient autonomy. The sustained improvements observed in this study suggest that APA may offer a practical complementary approach for long-term symptom management in cancer survivors living with chronic neuropathy.

### Limitations

Several limitations should be considered when interpreting these findings. First, there was no concurrent control group during the follow-up period because participants initially assigned to usual care crossed over to receive APA after the primary trial phase. Consequently, this analysis evaluates the sustainability of symptom improvements following APA rather than its long-term comparative effectiveness. Without an untreated comparison group, it is not possible to determine the extent to which maintained improvements were attributable to APA versus natural recovery, regression to the mean, placebo effects, or other unmeasured factors. In addition, follow-up was limited to three months after treatment completion, preventing conclusions regarding the durability of benefits beyond this period or the potential need for booster treatments.

Variability in cancer diagnosis, years since diagnosis, and baseline neuropathy severity may also have influenced symptom trajectories, and the study was not designed or powered to examine sustainability across these clinical subgroups. Exact time since cessation of neurotoxic chemotherapy was not available in the analytic dataset; therefore, years since diagnosis was reported as the available timing variable, although it is less directly relevant to CIN symptom persistence and recovery. In addition, inclusion of participants with pre-existing peripheral neuropathy that worsened after chemotherapy may have introduced heterogeneity in symptom etiology, and we cannot confirm that all neuropathy symptoms were attributable solely to CIN. Future studies should consider excluding documented non-chemotherapy-related neuropathies or stratifying analyses by pre-existing neuropathy status.

Attrition increased over time and was greater in the vAPA group than in the mAPA group, raising the possibility of retention bias if participants who experienced greater benefit were more likely to complete follow-up assessments. This concern is compounded by the relatively high proportion of missing data, particularly at the 2- and 3-month follow-up assessments. Although appropriate statistical methods were used to address missingness and minimize bias, the substantial reliance on imputed values limits confidence in the precision of the estimates and warrants cautious interpretation of the results. In addition, adherence to APA practice after intervention completion was not systematically monitored; participants may have continued APA or adopted other symptom-management strategies during follow-up, making it difficult to determine the extent to which sustained improvements were attributable to the intervention itself.

Outcomes were based primarily on patient-reported measures, which are essential for assessing symptom burden and functional impact but may be influenced by recall bias, participant expectations, and social desirability. Although objective assessments, including quantitative sensory testing and other functional measures, were collected as part of the parent trial, they were beyond the scope of the present follow-up analysis. Consequently, this study cannot determine whether the sustained patient-reported improvements corresponded to measurable neurological or functional changes. Future analyses integrating both objective and patient-reported outcomes may provide a more comprehensive understanding of the long-term effects of APA.

Finally, because participants volunteered for a behavioral symptom-management study, they may differ from the broader population of cancer survivors with CIN in motivation, health literacy, or attitudes toward complementary therapies, potentially limiting generalizability. Despite these limitations, the findings provide preliminary evidence supporting the sustainability of APA-related improvements in CIN symptoms and physical function. Larger studies with longer follow-up periods, objective outcome measures, systematic monitoring of post-intervention behaviors, and appropriate comparison groups are needed to confirm and extend these findings.

### Conclusion

In conclusion, improvements in CIN severity and CIN interference following a four-week APA intervention were maintained for up to three months after treatment completion. The persistence of these benefits after the end of active treatment suggests that APA may offer a sustainable, patient-centered approach for managing chronic CIN symptoms. The findings are consistent with prior APA studies in chronic pain populations and parallel evidence from auricular acupuncture interventions for diabetic peripheral neuropathy, both of which have demonstrated sustained improvements in neuropathic symptoms and functioning. However, the absence of a long-term control group and other methodological limitations warrant cautious interpretation of the findings. Future studies incorporating longer follow-up periods, appropriate comparison groups, objective outcome measures, and more diverse patient populations are needed to further evaluate the durability of APA-related improvements and determine its role in long-term survivorship care.

## Supplementary Material

supplementary material

**Supplementary Information** The online version contains supplementary material available at https://doi.org/10.1007/s11764-026-02104-3.

## Figures and Tables

**Fig. 1:**
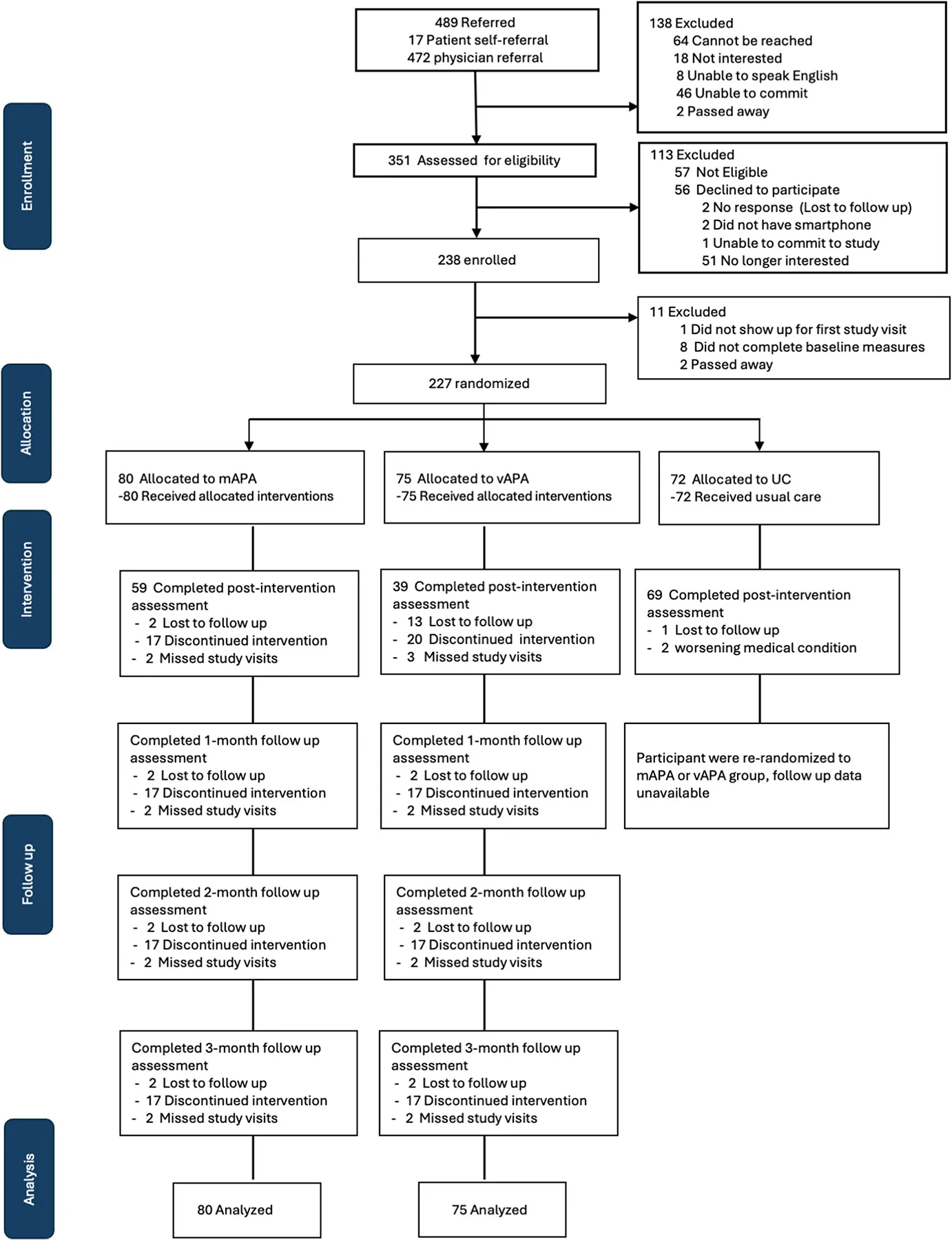
CONSORT diagram of study participation. Note: APA = auricular point acupressure; CI*N* = chemotherapy-induced neuropathy; CONSORT = Consolidated Standards of Reporting Trials; mAPA = mobile-supported auricular point acupressure; UC = usual care; vAPA = virtual auricular point acupressure

**Fig. 2:**
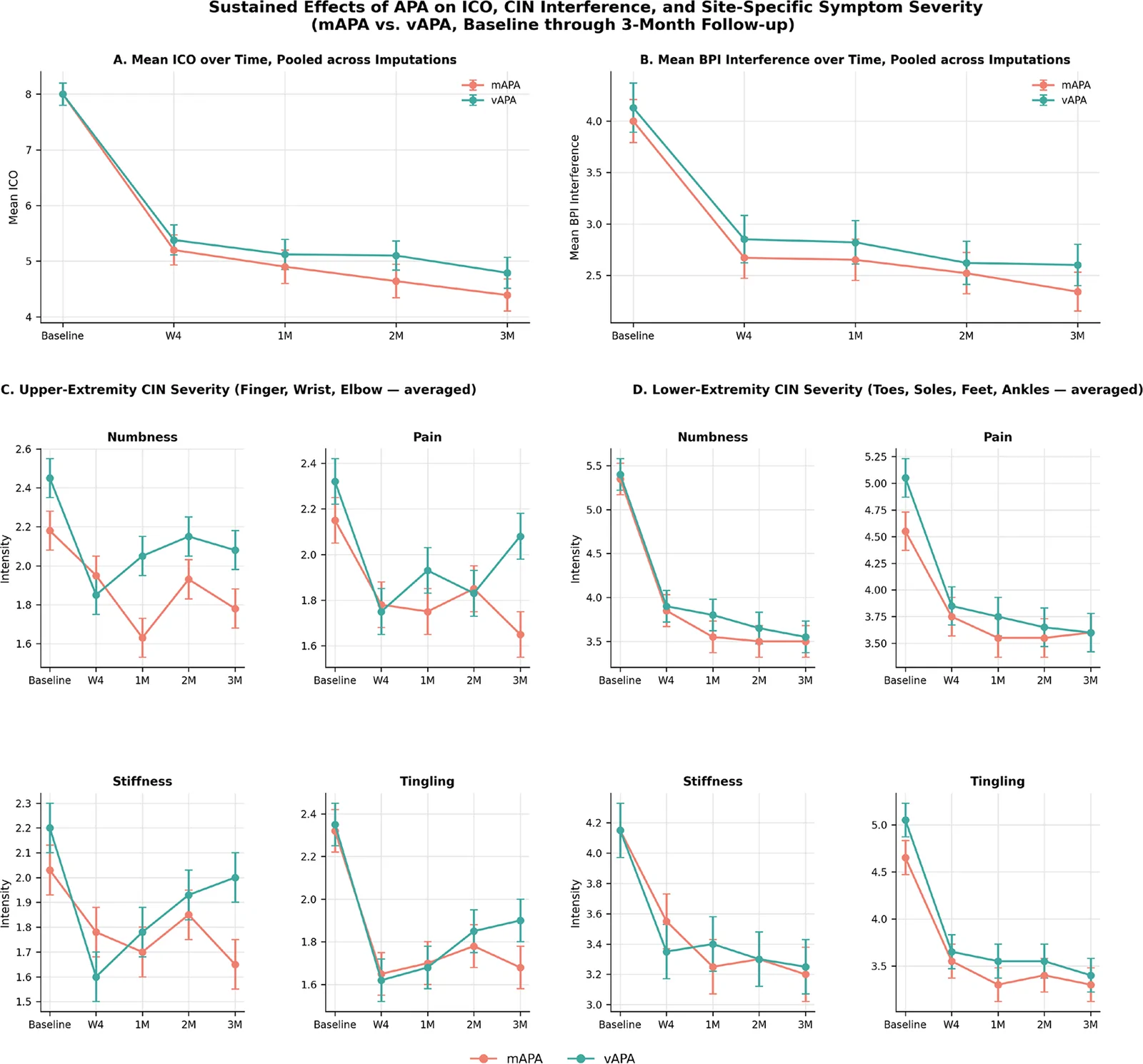
Sustained effects of APA from baseline through 3-month follow-up: (**A**) individualized composite outcome (ICO), (**B**) CIN interference, (**C**) upper-extremity symptom severity, and (**D**) lower-extremity symptom severity. Note: APA = auricular point acupressure; CI*N* = chemotherapy-induced neuropathy; ICO = individualized composite outcome; mAPA = mobile-supported APA; vAPA = virtual APA

**Table 1:** Demographics and baseline characteristics of study participants (*N* = 155)

Characteristics				mAPA (*N* = 80)	vAPA (*N* = 75)	*p* value
**Age (Mean (SD) (Range))**				65.10 (9.89) (35, 84)	62.47(12.72) (27, 87)	0.14
**Gender (n, %)**	Female			49 (60.8%)	42 (56.0%)	0.62
	Male			31 (39.2%)	33 (44.0%)	
**Race (n)**	Black			22	18	0.88
	White			52	51	
	Others			6	6	
**Education (n)**	High school, technical/vocational, or less			9	17	0.19
	Some college			15	17	
	College graduate			27	20	
	Post-graduate degree			29	21	
**Marital status (n)**	Currently married or living with a partner			52	46	0.59
	Separated, Divorced, Widowed			22	21	
	Never married			5	8	
**Cancer site (n)**	Breast			15	16	0.27
	Thoracic (lung, thyroid)			3	0	
	Gastrointestinal (rectal, pancreatic, liver)			23	27	
	Genitourinary (prostate, bladder, kidney)			7	5	
	Gynecologic (endometrial, ovarian)			6	2	
	Dermatologic (Melanoma)			0	1	
	Hematologic (Leukemia, Lymphoma)			1	4	
	Others			24	18	
	missing			1	2	
**Years Since Diagnosis (Mean (SD))**				4.69 (7.46)	3.68 (4.50)	0.81
**Use of Medication and/or Supplement**		For Pain (n, %)	Prescription	37, 46.2%	36, 48.0%	0.95
			OTC	20, 25.0%	15, 20%	0.58
			Supplement	8, 10%	11, 14.7%	0.54
		For Sleep (n, %)	Prescription	10, 12.5%	17, 22.7%	0.15
			OTC	6, 7.5%	4, 5.3%	0.75
			Supplement	9, 11.2%	7, 9.3%	0.90
**Charlson Comorbidity Index (Mean (SD))**				5.70 (3.28)	5.34 (3.56)	0.38
**Baseline Symptoms**						
**Neuropathy**	**Severity (ICO)**			8.01 (1.68)	8.00 (1.81)	0.89
	**Interference**			4.52 (2.44)	4.73 (2.36)	0.60
**Functional Status**		**FACT/GOG-Ntx (mean (SD))**		21.06 (7.60)	20.66 (7.09)	0.73
		**ECOG (n)**	0–1 = Good Performance	58	60	0.27
			≥2 = Impaired/frail	21	13	

Values are presented as mean (SD) for continuous variables and n (%) for categorical variables. *APA* auricular point acupressure, *CIN* chemotherapy-induced neuropathy, *ECOG* Eastern Cooperative Oncology Group, *FACT/GOG-Ntx* Functional Assessment of Cancer Therapy/Gynecologic Oncology Group–Neurotoxicity, *ICO* individualized composite outcome, *mAPA* mobile-supported auricular point acupressure, *OTC* over the counter, *SD* standard deviation, *vAPA* virtual auricular point acupressure

**Table 2:** Within-group changes in ICO and CIN interference scores during post-intervention follow-up relative to baseline

Follow-up Time	mAPA Estimate (95% CI)	*p* value	vAPA Estimate (95% CI)	*p* value
**ICO**				
1 Month	−3.10 (−3.78, −2.41)	< 0.001	−2.88 (−3.67, −2.08)	< 0.001
2 Month	−3.36 (−4.15, −2.57)	< 0.001	−2.90 (−3.63, −2.17)	< 0.001
3 Month	−3.61 (−4.42, −2.79)	< 0.001	−3.21 (−4.04, −2.38)	< 0.001
**CIN Interference**				
1 Month	−1.30 (−1.80, −0.80)	< 0.001	−1.18 (−1.68, −0.68)	< 0.001
2 Month	−1.36 (−1.85, −0.86)	< 0.001	−1.22 (−1.76, −0.67)	< 0.001
3 Month	−1.60 (−2.10, −1.10)	< 0.001	−1.28 (−1.84, −0.73)	< 0.001

Negative estimates indicate reductions in CIN severity/interference (improvement) relative to baseline. Individual upper- and lower-extremity symptom trajectories (numbness, pain, stiffness, tingling) are presented in Fig. 2

## Data Availability

The datasets generated and analyzed during the current study are available from the corresponding author upon reasonable request. Requests for access will be reviewed in accordance with institutional requirements, participant confidentiality protections, and applicable data-sharing policies.
