# Supplementary material for: Sustained effects of auricular point acupressure on chemotherapy-induced neuropathy: a randomized controlled trial follow-up study
Source: J Cancer Surviv. Author manuscript; Available in PMC 2026 Oct 14. (PMC13662696; doi:10.1007/s11764-026-02104-3)
Supplement: Supplementary file 1 [file NIHMS2213947-supplement-supplementary_material.docx]

**Supplementary**

**Table . Within-group changes in ICO and BPI CIN interference scores during post-intervention follow-up relative to baseline, using complete cases**

| **Outcome** | **Follow-up Time** | **mAPA Estimate (95% CI)** | **p value** | **vAPA Estimate (95% CI)** | **p value** |
| --- | --- | --- | --- | --- | --- |
| **ICO** | 1 Month | -3.03 (-3.62, -2.44) | <0.001 | -2.68 (-3.52, -1.83) | <0.001 |
|  | 2 Month | -3.43 (-4.13, -2.73) | <0.001 | -2.55 (-3.16, -1.93) | <0.001 |
|  | 3 Month | -3.59 (-4.36, -2.83) | <0.001 | -3.55 (-4.42, -2.68) | <0.001 |
| **CIN Interference** | 1 Month | -1.08 (-1.66, -0.50) | <0.001 | -1.36 (-1.98, -0.74) | <0.001 |
|  | 2 Month | -1.22 (-1.76, -0.67) | <0.001 | -1.52 (-2.20, -0.84) | <0.001 |
|  | 3 Month | -1.54 (-2.17, -0.91) | <0.001 | -1.66 (-2.34, -0.98) | <0.001 |
